# Transfer Learning in ECG Classification from Human to Horse Using a Novel Parallel Neural Network Architecture

**DOI:** 10.1038/s41598-019-57025-2

**Published:** 2020-01-13

**Authors:** Glenn Van Steenkiste, Gunther van Loon, Guillaume Crevecoeur

**Affiliations:** 1 Department of large animal internal medicine, Ghent University, Ghent, 9000 Belgium; 2 Department of Electromechanical, System and Metal Engineering, Ghent University, Ghent, 9000 Belgium; 3 Core Lab EEDT-DC, Flanders Make, Belgium

**Keywords:** Machine learning, Cardiology, Computer science

## Abstract

Automatic or semi-automatic analysis of the equine electrocardiogram (eECG) is currently not possible because human or small animal ECG analysis software is unreliable due to a different ECG morphology in horses resulting from a different cardiac innervation. Both filtering, beat detection to classification for eECGs are currently poorly or not described in the literature. There are also no public databases available for eECGs as is the case for human ECGs. In this paper we propose the use of wavelet transforms for both filtering and QRS detection in eECGs. In addition, we propose a novel robust deep neural network using a parallel convolutional neural network architecture for ECG beat classification. The network was trained and tested using both the MIT-BIH arrhythmia and an own made eECG dataset with 26.440 beats on 4 classes: normal, premature ventricular contraction, premature atrial contraction and noise. The network was optimized using a genetic algorithm and an accuracy of 97.7% and 92.6% was achieved for the MIT-BIH and eECG database respectively. Afterwards, transfer learning from the MIT-BIH dataset to the eECG database was applied after which the average accuracy, recall, positive predictive value and F1 score of the network increased with an accuracy of 97.1%.

## Introduction

A cardiac arrhythmia is an abnormal impulse generation or an abnormal conduction of the impulse from the sinoatrial node in the heart^1^. An electrocardiogram (ECG) allows to detect these abnormalities non-invasively. Horses often present arrhythmias at rest and/or during exercise and arrhythmias can range from being clinically irrelevant to potentially life threatening^2–5^. At the same time, sudden death during exercise occurs at an up to 10 times higher ratio in horses compared to human athletes^6,7^. Looking at the causes of sudden death, a cardiovascular problem is found in 8.8% of the cases, but in 68% of the cases no pathology is found on autopsy and a fatal arrhythmia is proposed as the most likely cause of sudden death^6,8^. Arrhythmias in horses may not be associated with obvious clinical signs which alert the owner, and simple auscultation is not always reliable, which requires the recording of an electrocardiogram to make a diagnosis^9^. Also in human medicine the use of an ECG has been proven to be the way to go for pre-participation screening^10^. However ECG interpretation is time-consuming and requires expertise. Furthermore, intra- and interobserver agreement for recognition and classification of arrhythmias varies from good during rest to poor during exercise which currently limits the usage of ECGs in horses^11^. Computer aided signal analysis can eliminate both intra- as interobserver variability and can be done quicker and more cost effective when compared to human interpretation.

**Figure 1:**
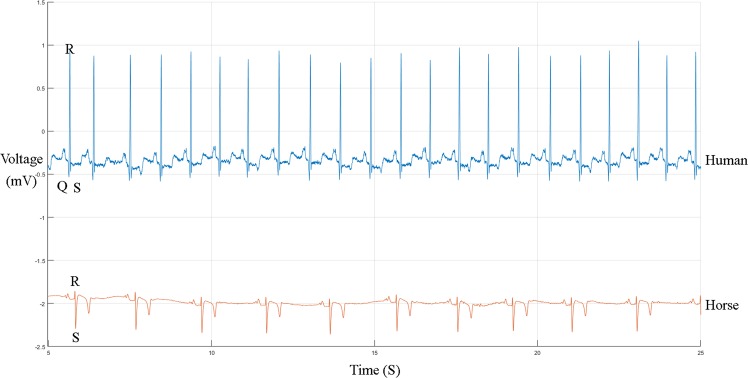
Humane (upper) and equine (lower) ECG at rest. Both ECGs are shown at the same paper speed and resolution. Note the difference in both morphology as basal heartrate.

Automated ECG interpretation is challenging since an ECG signal can vary between and within patients under different physical circumstances^12^. In order to perform diagnosis based on an ECG, the algorithm must be able to characterize and recognize ECG morphology and rhythm. For human medicine numerous algorithms have been proposed both for filtering, QRS detection and classification of ECGs, but for horses only one filtering algorithm, two QRS detection algorithms and no classification algorithms were described by the knowledge of the authors^3,13,14^. Hoofed animals, such as the horse, have a different nervous conduction system of the heart which results in a different ECG morphology as can been seen in Fig. 1^15^. One of the most striking differences between human and equine ECGs (eECGs) is that instead of having a large R peak, a large S peak is prominent in eECGs. Because there is currently a lack of horse-adapted software, equine ECGs are currently only manually analysed by a trained equine clinician or cardiologist^4,11^.

The aim of this study is to develop algorithms for an end-to-end system for beat-to-beat eECG analysis for horses based upon conventional ECG signal processing techniques along with state of the art deep learning techniques for feature extraction and classification. Since deep learning approaches require massive amounts of data to be trained, which is not available for eECGs, transfer learning will be applied in order to improve results for eECG classification. In most automated ECG interpretation studies, the authors concentrate on conventional machine learning approaches: pre-processing, feature extraction, feature reduction and feature classification^16^. The advantage of using deep learning above the conventional techniques is that the essential steps, namely feature extraction, feature selection and classification can be developed without explicit definition. Improvements in deep learning network architectures and more powerful computing hardware has recently increased the usage of deep learning networks for cardiac arrhythmia diagnosis in ECGs. Two of the most recent papers use short segments of ECG, 1 second and 2 seconds respectively, and use a residual convolutional neural network (CNN)^17,18^. Murugesan *et al*. also introduce the combined use of a long short memory (LSTM) block with a CNN showing improved results. The improved results can be explained because the relationship with surrounding heartbeats can yield important information which is used by the LSTM block. Recurrent neural networks have traditionally been used for time series processing, but they are resource intensive and recent results have shown that convolutional neural network approaches can outperform recurrent neural networks for tasks such as audio synthesis and machine translation^19^. Therefore we introduce a parallel network in this paper, with a separate pathway for feature extraction from both the individual ECG morphology and the temporal relationship. Despite the papers above achieving a high accuracy, sensitivity and positive predictive value (PPV), they lack the ability of providing the cardiologist an exact number of abnormal beats which is an important prerequisite in eECG analysis. Horses can have a heartrate up to 240 bpm (4 beats per second) and thus a segment of 1 or 2 seconds can include both normal and abnormal heartbeats simultaneously^20^. Another study by Isin and Ozdalili^21^ converts R-T segments to 256 × 256 × 3 images and uses the convolutional layers of a pre-trained alexNet^22^ as neural network architecture to extract the features, that on their turn are fed into a hidden layer after a principal component analysis is applied to reduce the number of features. Zihlman *et al*.^23^ and Rubin *et al*.^24^ both used a similar approach by converting the ECG to a spectrogram and feeding this into a CNN. Furthermore, Zihlman *et al*. also introduces a long short memory block between the convolutional layers and the hidden layers as network architecture. Lou *et al*.^25^ also converts the ECG to a spectrogram using a modified frequency slice wavelet transform before feeding the ECG to a stacked denoising auto-encoder for feature extraction, the result is then given to deep neural network for classification. Kachuee *et al*.^26^ and Kiranyaz *et al*.^27^ directly apply a CNN network using single unprocessed ECG beats as input to the network. The papers of Kiranyaz *et al*., Kachuee *et al*., Lou *et al*., Rubin *et al*. and Isin and Ozdalili have the advantage of providing beat per beat classification.

There are multiple annotated human ECG datasets available, with the Physionet MIT-BIH arrhythmia dataset as one of the most commonly used databases in studies^28,29^. Since for eECGs such datasets are lacking we use transfer learning from a network trained upon the MIT-BIH dataset to an own generated dataset for horses with 20.000 beats which were recorded in a clinical setting. Transfer learning for ECGs has already successfully been used in different studies for human ECG classification, but has not yet been applied between different species^17,21,27^.

## Results

### Datasets

Both the MIT-BIH and eECG dataset were split at random into a 60% training, 20% validation and 20% test dataset. The validation dataset was used as fitness value for the genetic algorithm and the validation and training dataset were used for training the final CNN. The test dataset was used for validation of the final trained CNN. Because both datasets are unbalanced in number of samples for each class, a resampling was applied for the training dataset. The MIT-BIH database has 15 different beat type classes, but since there is only limited knowledge and data about the different equine arrhythmias they were reallocated in the following 4 classes depending the underlying primary mechanism: Normal (N), atrial premature contraction (APC), ventricular premature contraction (VPC) or artefact (A).

The MIT-BIH dataset had 237704 beats for training the CNN, with 186874N, 11799 APC, 37153 VPC and 1878 A and 59427 samples for testing with 46841N, 2892 APC, 9232 VPC, 462A. The different classes were resampled to 24476 samples for each class for training, thus resulting in 97904 training samples in total. The eECG dataset was split in a training dataset of 21152 samples, with 15991N, 1087 APC, 3699 VPC and 375A which were resampled to 2393 samples of each class and 5288 samples for testing with 3972N, 255 APC, 971 VPC and 90A.

### Signal processing of the ECG signal

**Figure 2:**
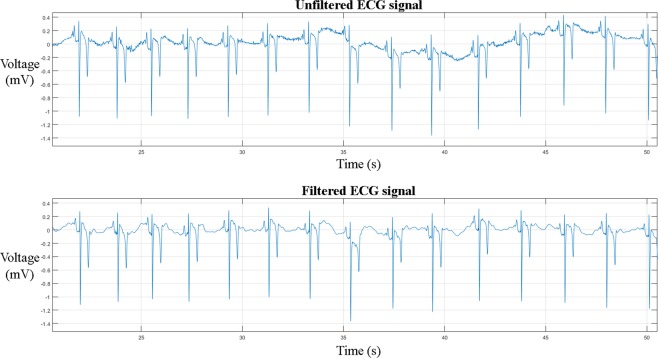
Equine ECG trace before (upper) and after (lower) filtering. A median filter has been applied in order to remove the low frequency baseline wander and a discrete wavelet filter was used to remove the high frequency noise.

The ECG signal was first pre-processed using a median filter to remove the baseline wander, followed by Discrete Wavelet transform to remove the remaining noise^30,31^. The result of the filtering is shown in Fig. 2. Next, S peaks in eECGs were detected based upon the modulus maxima of the Stationary Wavelet transform with a Symlet 4 wavelet and an adaptive threshold^31^. The detected beats were used for training and testing of the proposed CNN architectures.

The proposed QRS detection algorithm was compared to the popular Pan-Tompkins algorithm on 5 different 30 m eECG traces (10356 beats in total)^32^. The results of the comparison are shown in Table 1.

**Table 1:** Comparison of proposed QRS detection algorithm with Pan-Tompkins^32^ on 10356 equine QRS complexes.

Algorithm	False Positive (beats)	False Negative (beats)	Positive Predictive Value (%)	Recall (%)
Proposed	94	204	99.1	98.0
Pan-Tompkins	2156	737	81.5	91.5

### The ECG classification algorithm

**Figure 3:**
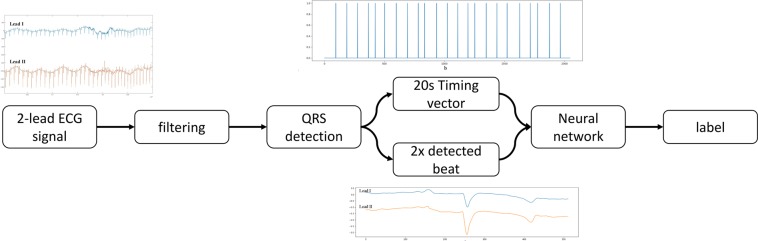
High level architecture of the complete proposed ECG processing method. Two raw ECG leads are filtered and QRS detection is performed on lead II. After QRS detection 500 points around the detected beat, both from lead I and lead II, are extracted in addition with a vector representation of all detected QRS complexes 10 s before and 10 s after each beat. These inputs are presented to the network that performs the classification into 4 classes.

**Figure 4:**
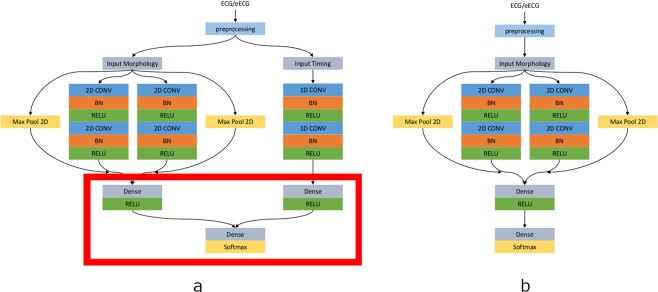
High level architecture of both proposed networks. Panel A shows the CNN with parallel timing pathway, panel B shows the CNN without timing pathway. The red rectangle highlights the layers that were retrained when only performing transfer learning on the top layers. CONV: convolutional block; BN: batch normalization; RELU: rectified linear activation unit; Max Pool 2D: Max pooling operation; Dense: fully connected layer; Softmax: Softmax activation block with the 4 different output classes.

**Figure 5:**
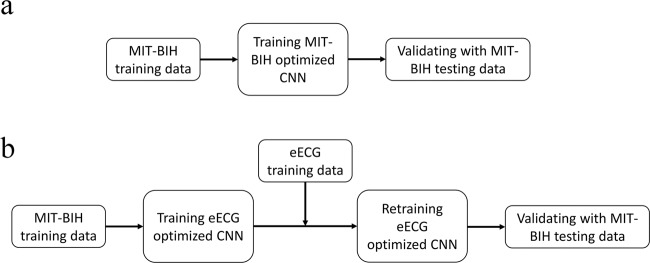
Training method for obtaining the evaluation metrics for the MIT-BIH dataset (Panel a) and the eECG dataset (Panel b). The MIT-BIH dataset is directly trained on the MIT-BIH training set (80% of total dataset) with the MIT-BIH optimized CNN parameters for the proposed CNN architectures. For the eECG dataset, the same MIT-BIH training set was used for obtaining the initial weights with the eECG optimized CNN parameters. Afterwards the network is retrained with the eECG training data.

The CNN were designed for a fixed network input of 2 × 500 data points for the morphological input and 2000 data points for the timing input. The high-level architecture of the complete proposed ECG processing method is shown in Fig. 3 and the high-level architecture of the proposed CNN’s is shown in Fig. 4. Weights were set using random weight initialization for the MIT-BIH dataset. For the eECG dataset, both randomly initialized weights and the weights from the pretrained CNN on the MIT-BIH dataset (transfer learning) were used for comparison. The used training method is shown in Fig. 5. A genetic algorithm (GA) was used to optimize the CNN parameters. The non-dominated sorting genetic algorithm II (NSGA-II) was used with a population size of 20 and optimized during 10 generations with the accuracy as fitness value and training was ended after 50 epochs or when the training accuracy stopped improving for 3 epochs^33^. The following parameters were optimized: number of filters, width of convolution and subsampling for each individual convolutional layer, number of neurons for each fully connected layer, L2 regularization and dropout used for training.

The details of the best network architecture calculated by the GA are given in Table 2. This network achieved an accuracy of 97.7% on the eECG validation dataset. The results of the different datasets and with or without transfer learning from the MIT-BIH dataset to the eECG dataset are given in Table 3 in addition with the results of training without resampling the dataset. The transfer learning was done using 2 different methods: the first method updated the weights of all the layers of the network during the training, while the second method only updated the weights of the fully connected layers. With the second transfer learning method the convolutional layers are used as pre-trained feature extractors while the fully connected layers are re-trained as classification layers. Results from the non-parallel network are shown in Table 4. It took around 13 h 46 m for running the GA, 20 m for training on the MIT-BIH dataset and 4 m 30 s for training on the eECG dataset for the entire network and 18 s for only retraining the top fully connected layers. In order to evaluate the performance of our proposed architecture on the eECG dataset, both the resampled MIT-BIH and eECG dataset were trained and evaluated with the deep CNN architecture proposed by Kachuee *et al*.^26^. Results of the deep CNN architecture shown in Table 5.

**Table 2:** Results from the genetic algorithm after optimizing both on the MIT-BIH dataset and the eECG dataset.

	CNN 1	CNN 2	CNN timing
**MIT-BIH**			
Layer 1			
Subsampling	4	2	128
Convolution width	60	20	300
Filters	16	40	4
Layer 2			
Subsampling	16	16	8
Convolution Width	30	20	100
Filters	16	32	8
Neurons in fully connected layers	512		512
Neurons in final fully connected layer	32		
**eECG**			
Layer 1			
Subsampling	8	2	32
Convolution width	10	30	50
Filters	16	8	64
Layer 2			
Subsampling	4	16	16
Convolution Width	50	20	30
Filters	64	64	4
Neurons in fully connected layers	512		512
Neurons in final fully connected layer	32

**Table 3:** Scores for the parallel network for different datasets, testing methods with or without resampling the datasets and different transfer learning techniques.

Class	Recall (%)	Positive Predictive Value (%)	F1-score (%)	Accuracy (%)
**Scores with resampling**				
*MIT-BIH*				97.7
N	99.4	97.3	98.4	
APC	70.1	94.8	80.6	
VPC	93.6	94.8	80.6	
A	81.9	90.6	86.0	
*eECG without transfer learning*				92.6
N	99.2	81.7	95.2	
APC	43.3	95.7	59.5	
VPC	97.7	96.2	96.9	
A	81.7	91.5	96.2	
*eECG with transfer learning on the entire network*				97.1
N	99.5	97.1	98.3	
APC	73.2	95.7	81.6	
VPC	97.1	97.8	97.4	
A	87.8	87.8	87.8	
*eECG with transfer learning on the top layers*				86.4
N	99.0	83.7	90.7	
APC	41.6	85.5	55.4	
VPC	75.4	97.5	84.9	
A	85.4	90.9	88.1	
**Scores without resampling**				
*MIT-BIH*				98.2
N	98.2	99.2	99.1	
APC	90.2	88.3	89.2	
VPC	97.8	98.2	97.6	
A	91.8	89.6	87.0	
eECG with transfer learning				98.2
N	99.0	99.1	99.0	
APC	90.9	92.6	91.7	
VPC	97.8	97.8	97.8	
A	92.2	81.5	85.1

**Table 4:** Scores for the CNN architecture without parallel timing input.

Class	Recall (%)	Positive Predictive Value (%)	F1-score (%)	Accuracy (%)
**Scores with resampling**				
*MIT-BIH*				96.7
N	99.7	96.8	98.2	
APC	69.0	94.4	79.7	
VPC	95.3	96.8	96.1	
A	82.0	89.4	85.5	
*eECG with transfer learning on the entire network*				91.9
N	99.5	90.5	94.7	
APC	43.5	96.7	59.1	
VPC	96.9	96.5	96.7	
A	81.0	90.0	85.3

**Table 5:** Scores for the deep CNN architecture proposed by Kachuee *et al*.^26^.

Class	Recall (%)	Positive Predictive Value (%)	F1-score (%)	Accuracy (%)
**Scores with resampling**				
*MIT-BIH*				95.9
N	99.3	96.3	97.8	
APC	68.5	91.4	78.3	
VPC	92.3	96.0	94.1	
A	77.3	86.3	81.5	
*eECG without transfer learning*				92.0
N	99.1	91.3	95.0	
APC	46.5	92.2	61.8	
VPC	96.0	96.1	96.0	
A	78.5	88.3	84.5	
*eECG with transfer learning on the entire network*				93.8
N	98.6	93.8	96.2	
APC	56.6	85.5	68.1	
VPC	94.1	96.9	95.4	
A	75.8	87.6	81.7	

## Discussion and Conclusion

Optimization of neural network hyperparameters is a time intensive task that can consume multiple weeks of computing time in order to combine all possible (realistic) parameters. Here we used a GA for optimization of the CNN parameters, which only consumed 13 h 46 m of computing time in order to run once without human interaction. The GA also achieved a good network reduction with relatively small network dimensions as can be seen in Table 2.

Since there are no published results of QRS detection algorithms on eECGs, we validated our proposed QRS detection algorithm against the Pan-Tompkins algorithm^32^. This algorithm is the most common used algorithm in literature for QRS detection on human ECG traces. As shown in Table 1 the Pan-Tompkins algorithm has a lower positive predictive value and sensitivity in comparison with our proposed algorithm. The high number of false positive results, and thus low positive predictive value, of the Pan-Tompkins algorithm is mainly explained due to the detection of the high T-wave as a QRS complex. In certain circumstances the detection of the T-wave obstructed the detection of the actual QRS complex, especially during higher heartrates during exercise, which explains the higher number of false negative results. Most false positive detected beats of the proposed algorithm were high frequency and high amplitude artefacts on the ECG trace which also induced a short blanking period due to the adaptive threshold of the algorithm. This blanking period accounts for most of the false negative beats of the proposed algorithm. The higher number of false positives would have implied a higher number of A, namely more T waves that would have been detected as QRS complexes, in the eECG dataset and thus a better balance of the different classes since there are currently only 465A in the eECG dataset or 2.2% of the total number of beats. However in combination with the lower sensitivity this would have resulted in a lower number of total true positives and therefore have an impact on the 1D timing vector which would be less representative for the relative timing of each QRS around the detected beat. If the 1D timing vector would include more artefacts and/or lesser QRS complexes this could possibly affect the usefulness of the 1D timing vector for the overall classification algorithm.

Currently there are no published equine ECG classifications methods and/or published datasets, therefore it is difficult to benchmark the performance of the proposed method for equine ECGs. The intra- and interobserver agreement has been suggested to be similar as for human ECGs, but the accuracy of the individual equine physiologist has not yet been studied^11^. When comparing with the numerous described algorithms for human ECG classification, our proposed method shows good classification results with an accuracy of 97.7% for the MIT-BIH database, with an even a higher accuracy of 98.2% when not applying resampling to the training dataset. However it should be noted that direct comparison between publications is difficult due to the different metrics, classes and datasets that are used for evaluating the classification performance^14^. In addition, accuracy alone is not the most optimal measurement of performance for ECG classification due to the imbalanced nature of ECG data which is illustrated here with a 99:1 ratio for the N:A classes in the MIT-BIH training dataset and a 43:1 ratio for the N:A classes in the eECG dataset. Since neural networks are even more vulnerable for overfitting when the dataset is imbalanced we used a resampling technique for the training data in order to achieve better classification results for the individual classes as has been shown before^23^. As can be seen in Table 3, the resampling had a negative effect on both the accuracy and individual scores, which is in contradiction to previous publications. The average recall, PPV and F1-score decreased from 94.7%, 93.8% and 93.2% to 86.3%, 94.5% and 90.5% for the MIT-BIH database and changed from 95.0%, 92.7% and 93.4% to 89.4%, 94.3% and 91.8% for the eECG dataset. A higher accuracy without resampling can be expected due to overfitting of the neural network on the majority class, the decrease of the individual scores however is unexpected. The effect might be explained due our resampling technique that noticeably decreases the total number of training samples, from 237704 to 97904 for the MIT-BIH dataset and from 21152 to 9572 for the eECG dataset. In addition to the decrease of the total number of samples the network can be trained on, and thus loss of information for the network, samples of the smallest class are copied multiple times and overfitting for this class can be expected. We tested both theories by undersampling to even lower numbers per class, which even further lowered the overall accuracy, and oversampling to higher numbers per class which slightly increased the overall accuracy and individual scores, but never exceeded the scores without resampling. This indicates that no extra information was learned by the network by copying the smallest classes, but this increased the required learning time with 50%. Another method to decrease overfitting and exploit the entire dataset is ensembling^23^. Multiple trained networks of the same type are used which are trained and validated, with early stopping, on different but overlapping parts of the dataset, the individual predictions are combined by majority voting. We applied this technique on our datasets with the parallel network for our production model with 5 trained networks and achieved an increased accuracy of 99.1% for the MIT-BIH dataset and 98.7% for the eECG dataset with improved (1–3%) performance metrics for all classes. Further improvements could be achieved by creating larger ensembles.

The results in Table 4 indicate that our proposed architecture with parallel timing input achieves a higher classification performance on all metrics, this effect remains for both the MIT-BIH and eECG dataset. These results reflect the clinical importance of the relative timing of each beat in the ECG trace that is also used by the cardiologist for the interpretation of the ECG. Other publications also acknowledge the importance of the temporal relationship by creating recurrent neural networks, for example by using LSTM blocks in combination with a CNN in 2 publications which had similar improvements in results as in our study by including the temporal relationship^17,23^. Larger CNN architectures with more layers or more parallel pathways, similarly to the inception block used in ECGNet, have been studied, but no further improvement in classification accuracy could be achieved^17^.

Because there are currently no other equine ECG classification methods published, we compared our method against the deep CNN network architecture of Kachuee *et al*.^26^. In order to validate our implementation of the deep CNN architecture, the deep CNN was also trained and validated on the MIT-BIH dataset as described in the paper. Since our implementation only use 4 classes instead of the 5 classes described in the original paper, our accomplished accuracy was slightly higher (95.9% vs. 93.4%) in comparison with the achieved accuracy in the paper as can be seen in Table 5. However the accuracy on all datasets remained lower in comparison with our proposed parallel CNN architecture.

When trained on the eECG dataset, the proposed network achieved a low accuracy of 92.6% when using randomly initialised weights. The results remained similar for both training with and without a resampled dataset, especially the recall and F1-score for the APC remained very low. Because the small and imbalanced dataset, the network is very vulnerable to overfitting despite the use of L2 regularisation and dropout layers. By using transfer learning from the similar but larger MIT-BIH dataset a significant improvement of accuracy and average recall, PPV and F1-score from 92.6%, 80.4%, 93.7% and 84.5% to 97.1%, 89.4%, 94,3% and 91.8% could be achieved due to the improved feature extraction of the CNN. However when the CNN layers were used a feature extractor and only the top classification layers were retrained for classification of the eECG dataset, the achieved average accuracy was even lower in comparison without transfer learning. This indicates that the features extracted from the MIT-BIH dataset, however being a good initial weight, cannot be directly applied to the eECG dataset without updating the weights of the acquired features. Transfer learning has been applied before for ECGs, both between datasets and for patient specific holter ECG interpretation, but by the knowledge of the authors never between species^17,25,34^. For the future, an improved, larger and better balanced, dataset should be used with the transfer learning for achieving even higher improvement of classification accuracy of eECGs with the current parallel network.

We propose two novel CNN architectures for ECG specific beat-to-beat classification using the beat morphology on 2 leads and a graphical representation of the timing of each beat. An equine specific filtering and QRS detection algorithm is proposed and validated against the popular Pan-Tompkins QRS detection algorithm. The classification model performance was validated on a human and an own-made equine dataset with and without resampling of the dataset for equally balanced classes. Transfer learning was used from the human to the equine dataset for improved classification performance for all classes of the equine dataset. Our results on both datasets were compared against the deep CNN network architecture of Kachuee *et al*.^26^. A genetic algorithm was used for successful optimization of the CNN parameters. By the best of the authors’ knowledge this is the first equine specific ECG classification algorithm and this algorithm may help to improve diagnosis of possible life-threatening arrhythmias in horses. Applying the architecture to larger (equine) ECG datasets for improved classification performance and extending the number of classes for classification are interesting pathways to be explored in the future.

## Methods

Procedures on animals were approved by the Ethical Committee, Faculty of Veterinary Medicine, Ghent University (EC2016/35). All experiments were performed in accordance with the relevant guidelines and regulations.

### Dataset description

The MIT-BIH arrhythmia database is one of the most used arrhythmia databases for research in ECG signal processing^28,35^. It contains 48 half-hour annotated, two-channel (lead II and modified V1, V2, V3, V4 or V5 leads) ambulatory ECG recordings, obtained from 47 patients sampled at 360 Hz per channel.

Because no datasets are available for eECGs we collected and annotated a dataset of 26.440 beats from 15 horses. Seven horses were used in a previous pacing study, in which ectopic depolarisations were introduced in the atrium and ventricle with electrical stimulation of the heart, resulting in an exact annotation of each beat^36^. The remaining 8 eECGs were selected from clinical cases based upon a high frequency of ectopic beats in order to include more clinical realistic timings of premature beats compared to the paced beats. Two veterinarians with experience in cardiology annotated these recordings using a Python based ECG annotation tool designed for this work. All eECGs were recorded at 500 Hz using the modified base-apex electrode configuration^37^. Because the eECGs were recorded at 500 Hz, the MIT-BIH dataset was upsampled in order to match eECGs. The ECG was subsampled to 100 Hz for the timing vector in order to reduce the memory and computational cost, the resolution remained 500 Hz for the morphology input. Lead I and II were used for the algorithm from the eECG dataset.

The median number of samples of each class was calculated for each dataset and the samples of each training class were randomly under- or oversampled with replacement in order to acquire the median number of samples for each class. This was done using the imbalanced-learn toolkit v 4.3^38^.

### eECG specific pre-processing

Baseline wandering is the low frequency noise typically induced by movement and breathing of the horse and can induce large differences between the amplitudes of the beats. A median filter can be used to remove this artefact without chancing or disturbing the characteristics of the waveform. The original eECG signal was processed with a median filter of 200 ms width in order to remove P waves and QRS complexes, followed by a median filter of 600 ms width to remove T waves^30^. The signal resulting from the second filtering contains the baseline of the ECG signal and is subsequently subtracted from the original ECG signal to obtain an ECG signal without baseline wandering.

After removing the baseline wandering, the resulting ECG signal still contains other residual noise in the higher frequency range. In this study we used Discrete Wavelet transform using a Coiflet 2 wavelet to filter the signal^31^. The signal was first decomposed into several subbands by applying Wavelet Transform with a Daubechies mother wavelet of order 8, since this has been proven to be the most appropriate wavelet basis function for denoising ECGs^39^. Next, all values below a certain threshold were set to 0, after which the signal was reconstructed. Using these pre-processing methods high frequency components of the ECG signal decrease as lower details are removed from the original signal. An example of an ECG trace filtered by a subsequent median and Discrete Wavelet filter is shown in Fig. 2.

### Detection of S peak in the eECG

The detection of the S peak is based upon the modulus maxima of the Stationary Wavelet transform using a Symlet 4 wavelet^31^. A Symlet 4 wavelet was used to decompose the signal since this best represents the shape of a physiologic equine QRS complex. For detection of the S peak, only the quadrated values of coefficient detail scale 4 (cd4²) are used for applying a search for a maxima modulus line exceeding an adaptive threshold t_QRS_^40^. t_QRS_ is calculated proportional to a rolling mean over 200 relative maxima of the selected detail coefficients. So for each 200 local maxima we take:

1 $${t}_{QRS}=\frac{1.25}{200}\,\mathop{\sum }\limits_{k=0}^{199}\,cd{4}_{n-k}^{2}$$

If multiple values exceed t_QRS_ within 200 ms, the largest value is selected as the QRS complex. A value of 200 ms is selected because this corresponds to the shortest duration of the effective refractory period of the action potential in horses with a 99% confidence interval^41^. For this study, detection occurred on lead II and a sample was taken from 0.5 s before and after each detected S peak on lead I and II for input to the CNN.

Since there are no published results from eECG specific QRS detection algorithms, the proposed method was compared against the Pan-Tompkins algorithm^32^. This is one of the most common used QRS detection algorithms. Both the Pan-Tompkins algorithm and the proposed algorithm were implemented in Matlab 2018b (MathWorks). Both algorithms were run on 30 m eECGs of 5 different horses. The results were manually checked for correctness.

### Parallel convolutional neural network architecture for eECG and ECG classification

Convolutional layers of different size have been shown to learn distinct feature representations and thus combining these features will provide better feature representation compared to one single filter^17,42^. Based on this, the input layers for the filtered signal is connected to 2 parallel paths with each identical architectures but different parameters. We propose a CNN architecture with two parallel input layers: one layer ingests the filtered signal of the detected beats of lead I and II in a 2 × 512 matrix while a second layer ingests a 1D vector (1 × 2048 samples) representing the relative timing of each detected beat in a 20 s segment, using 1 for a detected beat and 0 otherwise. Sample inputs are shown in Fig. 3. By doing so, the network is forced to learn both features that represent the morphology, the filtered signal input, and the temporal relationship of the detected beat, the 1D vector, compared to the surrounding beats. Both of these features are essential for correct ECG interpretation. Most approaches for individual beat classification only process the individual beat, without information of the surrounding ECG trace. While some ECG classification strategies involve the processing of longer traces with multiple beats, thus extracting possible temporal relationship features, they do not offer the benefit of beat-to-beat classification. Each parallel path consists of 2 2D convolutional layers, each followed by a batch normalization layer, a rectified linear unit activation (ReLU) block and a dropout layer^43–45^. The CNN layer performs convolution with a convolution width kernel size of 2*convolution width with 1*subsampling strides, so no interaction exists between both leads, the convolution width and subsampling stride are shown in Table 2 for each network. Zero-padding was used after convolution in order to keep the temporal order of the input signal. The batch normalization layers normalize the learned features of the previous CNN layer resulting in reduced overfitting and improved learning speed. The ReLu layer permits to add a non-linearity to the network which favours a deeper representation. The dropout layer improves the generalization capabilities of the network and avoids co-adaptation by randomly setting a fraction (=1 − p) of the neurons to zero. Based upon the residual like architecture of Rajpurkar *et al*.^18^, a residual connection is used within each path in a similar manner to those found in the Residual Network architecture^46^. This shortcut connection subsamples the input using a Max Pooling operation with the same subsample factor as the combined convolutional layers of that path^47^. The 2 parallel paths are flattened, concatenated and connected to a fully connected layer with a ReLU layer.

A separate parallel pathway processes the 1D vector with the relative timing information of the detected beat. The path consists of two 1D convolutional layers each followed by a dropout layer, no improvements in overall accuracy could be achieved by adding batch normalization blocks and residual connections as was done for the morphology paths. The last dropout is also connected to a fully connected layer with a ReLU layer. The outputs of the fully connected layers of the morphology and timing pathway are concatenated and connected to a final fully connected layer with soft-max activation in order to produce a distribution over the 4 output classes for each detected beat. The soft-max layer is used to perform closed-set identifications. The output of this layer is an integer label correlating to a predefined class. By adding a dedicated fully connected layer for each parallel pathway, the extracted features can be processed by a ‘specialized’ layer for each type of feature, timing or morphology, before feeding it into a general fully connected classification layer. Both a CNN with and without parallel pathway for timing were built and tested. The high-level architectures of both networks are shown in Fig. 4.

Since there are no other published equine ECG classification methods to compare against, the deep CNN architecture of Kachuee *et al*. was implemented and trained on our eECG dataset^26^. This architecture was chosen because of both the good accuracy on the MIT-BIH dataset and the ability to perform beat per beat classification. All deep CNN network parameters were set as described in the paper.

### Training method

The softmax cross entropy was used as loss function for training. The Adam optimizer with the default parameters described in the paper and with a learning rate of 0.0001 was used for updating the weights^48^. Dropout and L2 regularization was set to 0.2 and 0.001 respectively. Batch size was fixed to 500 and number of epochs to 20. The networks were build using Keras with Tensor Flow backend^49^.

The deep CNN was trained as described in the paper with the Adam optimizer, with the learning rate, beta-1, and beta-2 of 0.001, 0.9 and 0.999, respectively^26^. Learning rate is decayed exponentially with a decay factor of 0.75 every 10000 iterations. The MIT-BIH optimised CNN parameters were used for evaluating the performance on the MIT-BIH dataset, the eECG optimized CNN parameters were used for evaluating the performance on the eECG dataset. The training method is visualised in Fig. 5.

Transfer learning offers a useful solution for models were limited training data, a lack of expertise for training and moderate computer resources for training are available. In transfer learning a pre-trained network is used for a different task, with fine-tuning of some or all layers with the data from the different task. With a CNN, the convolutional layers can be reused as a feature extractor while the final layers (typically deep layers) can be retrained for a different classification task. Since the eECG dataset (26,440) is significantly smaller compared to the MIT-BIH ECG dataset (297,131 beats) transfer learning was applied from the MIT-BIH trained model to the eECG trained model. After training the complete model on the MIT-BIH ECG dataset, transfer learning to the eECG dataset was applied in two different ways:

- All weights of the pre-trained network are updated. After training the model on the MIT-BIH dataset all weights are reused as initial weights for training on the eECG dataset, so both the feature extractor (convolutional layers) and classification part (fully connected layers) are adapted to the eECG dataset.
- Using the pre-trained MIT-BIH network as a feature extractor. In this case all layers were frozen for training after training on the MIT-BIH dataset (i.e. the weights were not updated during training) except the fully connected layers. By doing so the pre-trained CNN acts as a feature extractor for the ECG and only the classification layers are retrained.

### Evaluation metrics

Standard metrics for classification tasks were used for evaluation: accuracy (ACC), Recall; positive predictive value (PPV) and F1-score, which are defined next:

2 $$\begin{array}{rcl}ACC & = & \frac{(TP+TN)}{(TP+TN+FP+FN)}\\ Recall & = & \frac{TP}{(TP+FN)}\\ PPV & = & \frac{TP}{(TP+FP)}\\ F1-score & = & \frac{2\,\ast \,R\,\ast \,PPV}{(R+PPV)}\end{array}$$

TP: true positive, TN: true negative, FP: false positive, FN: false negative, R: recall.

Each CNN was trained 3 times with identical initial parameters, the metrics were averaged over the results of these 3 trainings. The following tests were conducted:

- Each CNN (deep CNN, proposed CNN with and without parallel timing pathway) was tested with the resampled MIT-BIH and eECG dataset.
- The resampled eECG dataset was tested with both the network without transfer learning and with both transfer learning methods.
- In order to test the effect of the resampling, both the MIT-BIH and eECG dataset without resampling were used to train the parallel and deep CNN network with transfer learning from MIT-BIH to eECG.

## Data Availability

The datasets generated during and/or analysed during the current study are available from the corresponding author on reasonable request.
